# Stabilisation of p53 enhances reovirus-induced apoptosis and virus spread through p53-dependent NF-*κ*B activation

**DOI:** 10.1038/bjc.2011.325

**Published:** 2011-08-23

**Authors:** D Pan, L-Z Pan, R Hill, P Marcato, M Shmulevitz, L T Vassilev, P W K Lee

**Affiliations:** 1 Department of Microbiology and Immunology, Dalhousie University, Halifax, Nova Scotia, Canada B3H 4R2; 2 Discovery Oncology, Roche Research Center, Hoffmann-La Roche Inc., 340 Kingsland Street, Nutley, NJ 07110, USA; 3 Department of Pathology, Dalhousie University, Halifax, Nova Scotia, Canada B3H 4R2

**Keywords:** reovirus, Nutlin-3a, p53, NF-*κ*B, apoptosis

## Abstract

**Background:**

Naturally oncolytic reovirus preferentially kills cancer cells, making it a promising cancer therapeutic. Mutations in tumour suppressor p53 are prevalent in cancers, yet the role of p53 in reovirus oncolysis is relatively unexplored.

**Methods:**

Human cancer cell lines were exposed to Nutlin-3a, reovirus or a combination of the two and cells were processed for reovirus titration, western blot, real-time PCR and apoptosis assay using Annexin V and 7-AAD staining. Confocal microscopy was used to determine translocation of the NF-*κ*B p65 subunit.

**Results:**

We show that despite similar reovirus replication in p53^+/+^ and p53^−/−^ cells, stabilisation of p53 by Nutlin-3a significantly enhanced reovirus-induced apoptosis and hence virus release and dissemination while having no direct effect on virus replication. Enhanced apoptosis by Nutlin-3a was not observed in p53^−/−^ or p53 knockdown cells; however, increased expression of *Bax* and *p21* are required. Moreover, elevated NF-*κ*B activation in reovirus-infected cells following Nutlin-3a treatment was necessary for enhanced reovirus-induced apoptosis, as synergistic cytotoxicity was overcome by specific NF-*κ*B inhibitors.

**Conclusion:**

Nutlin-3a treatment enhances reovirus-induced apoptosis and virus spread through p53-dependent NF-*κ*B activation, and combination of reovirus and Nutlin-3a might represent an improved therapy against cancers harbouring wild-type p53.

Mammalian reovirus is a benign virus that has long been used as a model to study virus–host interactions. Reovirus was discovered to preferentially replicate in cancer cells and later shown to target cells with aberrant Ras-signalling pathways, suggesting that reovirus has the potential applicability as an oncolytic therapy (Hashiro *et al*, 1977; Duncan *et al*, 1978; Coffey *et al*, 1998; Strong *et al*, 1998). Indeed, *in vitro* and *in vivo* studies using human tumour xenografts in mice show that reovirus can induce regression of many cancers, including breast cancer, ovarian cancer and lymphoma (Wilcox *et al*, 2001; Alain *et al*, 2002; Hirasawa *et al*, 2002, 2003; Norman *et al*, 2002). Currently, reovirus (Reolysin) is entering phase III clinical trials in the United States and the United Kingdom for head and neck cancer (Bell, 2010).

Our group and others have reported that the extent of reovirus-induced apoptosis is significantly higher in Ras-transformed cells relative to untransformed cells (Smakman *et al*, 2005, 2006; Marcato *et al*, 2007; Shmulevitz *et al*, 2010). As reovirus-induced apoptosis plays an important role in virus cell-to-cell spread and tumour clearance, strategies that enhance apoptosis in cancer cells should promote reovirus oncolysis. Indeed, combining reovirus treatment with radiotherapy or chemotherapies has been shown to significantly enhance cancer cell apoptosis compared to single reagent treatment alone (Twigger *et al*, 2008; Pandha *et al*, 2009; Sei *et al*, 2009).

The tumour suppressor protein p53 plays a major role in dictating the fate of a cell upon exposure to stress or genomic damage, and mutant forms of this protein are found in over 50% of human cancers (Dey *et al*, 2008). A major effort in combating human cancer is therefore directed at restoring p53 tumour suppressor functions in cancers harbouring aberrant p53 response (Dey *et al*, 2008; Brown *et al*, 2009). An important negative regulator of p53 is the murine double minute 2 (MDM2) protein, which targets p53 for proteosomal degradation and which also inhibits p53 transcriptional activity. Nutlin-3a is the active enantiomer of Nutlin-3, a small molecular antagonist of MDM2. Nutlin-3a has been shown to induce p53 accumulation in human cancers that contain wild-type p53 and consequently promote cancer regression (Vassilev *et al*, 2004; Tovar *et al*, 2006).

Although the involvement of Ras signalling in reovirus oncolysis has been thoroughly investigated, the possible role of p53 in reovirus oncolysis remains relatively undefined (Huang *et al*, 2004; Kim *et al*, 2010). This study shows that reovirus replication, cell-to-cell spread and the extent of reovirus-induced apoptosis are comparable in p53 wild-type (p53^+/+^) human colon cancer HCT116 cells and their p53-null derivative (p53^−/−^). However, stabilisation of p53 by Nutlin-3a treatment significantly enhances reovirus-induced apoptosis and cell-to-cell spread in HCT116 p53^+/+^ cells as well as in a variety of human cancer cells containing wild-type p53. We demonstrate that this enhanced cytotoxicity corresponds with the elevated expression of p53 target genes and is dependent on NF-*κ*B activation. These studies suggest that combined reovirus and Nutlin-3a treatment could prove to be superior to single-agent treatment in cancers that retain p53 function.

## Materials and methods

### Cell lines, reovirus and reagents

HCT116 cells (parental p53^+/+^, p53^−/−^, PUMA^−/−^, Bax^−/−^, p21^−/−^, PUMA^−/−^p21^−/−^ and Bax^−/−^p21^−/−^) were a gift from Dr Bert Vogelstein (John Hopkins University, Baltimore, MD, USA); A549, U2OS and L929 cells were from American Type Culture Collection (Manassas, VA, USA).

Reovirus (Serotype 3-Dearing, T3D) was propagated in L-929 culture and purified as established procedures (Smith *et al*, 1969). Reovirus activity was determined by standard plaque titration.

Nutlin-3a was provided by Hoffmann-La Roche Inc. (Nutley, NJ, USA); RITA (*r*eactivation of p53 and *i*nduction of *t*umour cell *a*poptosis), Z-VAD(OMe)-FMK (ZVAD), Insolution NF-*κ*B activation inhibitor (**N**) and InSolution BAY 11-7082 (**B**) were purchased from Calbiochem (San Diego, CA, USA); propidium iodide (PI) from Sigma-Aldrich (St Louis, MO, USA); and soluble DR4, DR5 and anti-TRAIL antibody from R&D Systems (Minneapolis, MN, USA).

Antibodies against p53 (Do-I), *β*-actin, p65, PUMA, Bax and p21 were purchased from Santa Cruz Biotechnology Inc. (Santa Cruz, CA, USA).

### Immunostaining for NF-*κ*B p65 nuclear translocation

Cells grown overnight on gelatin-coated cover slides were treated with Nutlin-3a, reovirus or both. At 12-h post-infection (hpi), cells were fixed and then stained with anti-p65, anti-reovirus antibodies, followed by Alexa Fluor 488 F(ab′)_2_ fragment of goat anti-rabbit IgG (H+L), Cy3 AffiniPure Goat Anti-Mouse IgG (H+L) (Jackson ImmunoResearch Laboratories Inc., West Grove, PA, USA) and To-Pro-3 (Invitrogen Canada, Burlington, ON, Canada) for nuclear staining. Images were captured with a Zeiss LSM 510 laser scanning confocal microscope.

### Apoptosis assay, sub-G1 profiling and fluorescence-activated cell sorting analysis

Unless otherwise indicated, at 24 hpi, cells cultivated in the presence or absence of inhibitors were harvested and stained with Annexin V-FITC and 7-AAD (BD Pharmingen, Oakville, ON, Canada) according to the manufacturer's instructions. Cell death was quantified by flow cytometry using a FACScan flow cytometer (BD Biosciences, San Jose, CA, USA).

For sub-G1 profiling, cells were fixed with 70% ice-cold ethanol for 30 min, washed with PBS and then stained with PI staining buffer (50 *μ*g ml^−1^ of PI, 20 *μ*g ml^−1^ RNase A and 0.5% BSA) for 30 min. Two million cells were sorted and quantified by fluorescence-activated cell sorting (FACS) and analysed using WinMDI Version 2.8.

### Transfection and shRNA construct

Plasmids were transfected with Lipofectamine 2000 transfection reagent (Lipo) (Invitrogen) at an 1 : 3 ratio of DNA : Lipo according to the manufacturer's instruction. Specifically, 1.6 *μ*g of pcDNA3 (Invitrogen) or pcDNA3-p53 was transfected into HCT116 cells in the absence of AA. The same ratio of DNA : Lipo was used to transfect retroviral vector pSMP with either scrambled sequence or p53-specific shRNAmir sequence (V2HS_93615, Open Biosystems; Thermo Fisher Scientific Inc., Huntsville, AL, USA) into a packaging cell line as per the manufacturer's instructions and the resulting retrovirus supernatants were applied to A549 or U2OS cells with 2 *μ*g ml^−1^ Sequa-brene. Stable cells were selected by the addition of 2 *μ*g ml^−1^ puromycin at 48-h post-retrovirus.

### RNA extraction and real-time quantitative polymerase chain reaction

RNAs were extracted with TRIzol and PureLink RNA mini-column (Invitrogen). RNAs were reverse transcribed using the Superscript II reverse transcriptase kit (Invitrogen). QuantiFast SYBR RT-PCR kit (Qiagen, Mississauga, ON, Canada) and gene-specific primers (Supplementary Table S1) were used to quantify gene expression as per the manufacturer's instruction. Standard curves were generated to calculate relative level of mRNA and GAPDH is used as an internal control.

### Dual-luciferase assay

HCT116 cells (1 × 10^6^ cells/well in six-well plates) were transfected with 3.1 *μ*g pNF-kB-luc (Clontech Laboratories Inc., Mountain View, CA, USA) and 0.08 *μ*g pGL4.74 (Promega, Madision, WI, USA) 24 h before Nutlin-3a treatment. At 6-h post-transfection, one out of four cells were divided into 12-well plates and allowed to settle until 24-h post-transfection. Cells were then treated with vehicle (DMSO) or Nutlin-3a and at 6-h post-treatment infected by reovirus. Cells were lysed by passive lysis buffer (Dual luciferase assay kit; Promega) at indicated time and the collected lysates were flash–frozen and kept in −80 °C until use. Luciferase activity was measured by a GloMax Luminometer (Promega).

### Calculation of IC_50_

Promega CellTiter 96 AQ_ueous_ Non-Radioactive Cell Proliferation Assay (Promega) was used to determine the viability of reovirus- and/or Nutlin-3a-treated cells according to the manufacturer's instruction. Specifically, 1.5 × 10^4^ cells per well of HCT116 p53^+/+^ or p53^−/−^ cells were seeded onto 96-well plates at a volume of 100 *μ*l. Reovirus (serial dilution from an MOI of 100 to 1 × 10^−2^) was used to infect cells in triplicate wells after they were treated with 5 *μ*M Nutlin-3a for 6 h. At 48 hpi, 20 *μ*l of the MTS and PMS reagent mixture (20 : 1) was added to each well using multi-channel pipette and the plate was incubated at 37 °C for 1 h before being read at 492 nm by an ELISA microplate reader. IC_50_ was calculated using GraphPad Prism v.4.

## Results

### p53 stabilisation by Nutlin-3a enhances reovirus-associated cell death and release of progeny virions

**Figure 1:**
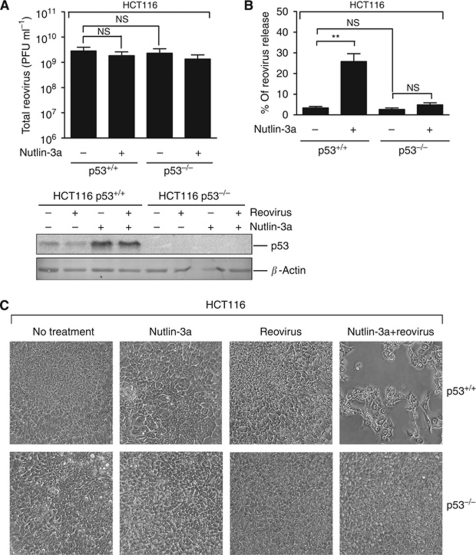
p53 stabilisation by Nutlin-3a enhances virus-associated cytotoxicity and release of progeny virions. (**A**) Upper panel: Human colon cancer HCT116 cells (p53^+/+^ and p53^−/−^) pretreated with 5 *μ*M Nutlin-3a for 6 h were infected at an MOI of 1. Total virus samples were collected at 18 hpi and quantified by plaque titration (±s.e.m., *n=3*). Lower panel: western blot analysis to determine levels of p53 upon reovirus or Nutlin-3 treatment at 24 hpi. (**B**) Percentage of reovirus released into the media was determined by dividing reovirus titres from infected cell media by the total reovirus titre (±s.e.m., *n=3*). (**C**) p53-dependent cytotoxicity enhancement caused by the combination of reovirus and Nutlin-3a. HCT116 p53^+/+^ or p53^−/−^ cells were mock infected or infected at an MOI of 1 in the presence or absence of 5 *μ*M Nutlin-3a. Images were taken at 24 hpi. Unless otherwise indicated, no treatment indicates that they were treated with dimethyl sulfoxide (DMSO, vehicle). Student's *t*-test was used to compare two groups of data; NS: not significant; ^**^P<0.001.

The prominent function of p53 in regulating apoptosis raised the possibility that p53 status might influence reovirus-mediated apoptosis. To address this question, we compared the efficiency of reovirus replication in HCT116 cells containing constitutively activated K-Ras (Fearon *et al*, 1990) and wild-type p53 (p53^+/+^) *vs* their p53-null isogenic derivative (p53^−/−^) cells (Bunz *et al*, 1998). By 18 hpi, total virus titres were comparable between HCT116 p53^+/+^ cells and HCT116 p53^−/−^ cells, indicating that deletion of p53 did not affect reovirus production (Figure 1A).

To test whether *accumulation/activation* of p53 can alter reovirus replication in cancer cells, HCT116 (p53^+/+^) cells were treated with Nutlin-3a. As expected, 5 *μ*M Nutlin-3a caused immediate (within 3 h) and significant p53 accumulation, and increased the expression of p53-regulated p21 and PUMA by 6 h post-treatment (Supplementary Figure S1A). Similarly, levels of p53 were significantly increased with the addition of Nutlin-3a in U2OS cells within 6 h (Supplementary Figure S1B). Cells were therefore treated with 5 *μ*M Nutlin-3a for 6 h before reovirus infection and total reovirus titres were measured at 18 hpi. Levels of p53 were markedly increased by Nutlin-3a treatment regardless of reovirus infection (Figure 1A). Moreover, p53 accumulation induced by Nutlin-3a treatment did not affect total reovirus production (Figure 1A). Overall, the extent of virus production in a single round of replication was impervious to the status of p53 in HCT116 cells.

Virus oncolysis depends not only on efficient virus production in cancer cells, but also on the efficient killing of infected cells, which facilitates both cytotoxicity and release of progeny virus for cell-to-cell spread. Experiments were therefore performed to determine if p53 affects reovirus-induced cytotoxicity and virus release. In the absence of Nutlin-3a, the titres of released (i.e., extracellular) reovirus were similar in p53^+/+^ and p53^−/−^ HCT116 cells, suggesting that cell death and subsequent virus release were unaffected by p53 deletion (Figure 1B). However, the titres of extracellular reovirus were significantly increased following Nutlin-3a treatment of infected p53^+/+^, but not p53^−/−^ cells (Figure 1B). The enhanced release of reovirus from infected p53^+/+^cells following Nutlin-3a treatment was also accompanied by a significant increase in cytotoxicity (Figure 1C). Cytotoxicity was dependent on productive reovirus replication, as cells treated with UV-inactivated reovirus and Nutlin-3a were relatively healthy (data not shown). Altogether, accumulation of p53 by Nutlin-3a in reovirus-infected cancer cells facilitates cell death, and hence release of progeny virions.

### Nutlin-3a significantly enhances caspase-dependent apoptosis of reovirus-infected cancer cells

**Figure 2:**
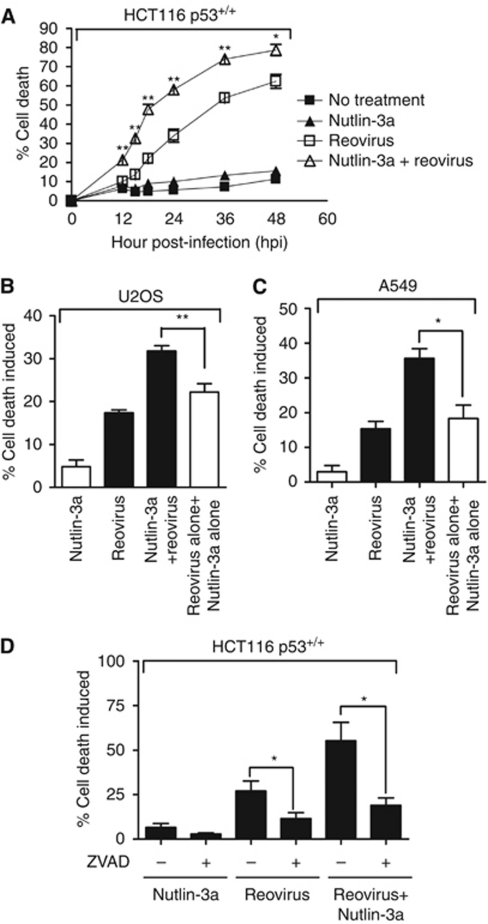
Nutlin-3a treatment induces significantly higher level of cell death in reovirus-infected p53^+/+^ tumour cells. (**A**) Percentage of cell death in HCT116 p53^+/+^ cells caused by reovirus, Nutlin-3a or the combination of the two quantified by Annexin V and 7-AAD staining. The apoptotic fraction was calculated as the sum of Annexin V-positive live cells and dead cells (upper and lower right quadrants). Samples were collected at 12, 15, 18, 24, 36 and 48 hpi. (**B** and **C**) Cell death induced by reovirus in the presence or absence of Nutlin-3a treatment in U2OS (*n*=3, ±s.e.m.) or in A549 (*n*=3, ±s.e.m.) cells. A549 and U2OS cells were infected at an MOI of 100 and 500, respectively. Samples were collected at 24 and 48 h, respectively, for Annexin V and 7-AAD staining. (**D**) Caspase inhibitor ZVAD blocked apoptosis induced by reovirus or the combination of Nutlin-3a and reovirus. Cells were treated with ZVAD (50 *μ*M) at the same time of Nutlin-3a treatment and throughout infection. Student's *t*-test was used to compare two groups of data; ^*^P<0.05; ^**^P<0.001.

The effects of Nutlin-3a on cytotoxicity of reovirus-infected cells were further characterised using Annexin V and 7-AAD staining to quantify the extent of early apoptosis and cell death. As shown previously (Tovar *et al*, 2006), Nutlin-3a alone induced minimal cell death (Figure 2A) in HCT116 cells. However, the percentage of cell death induced by reovirus was significantly enhanced by pretreatment with Nutlin-3a in HCT116 p53^+/+^ cells over the 48-h course of infection (Figure 2A). Treatment of cells with the inactive enantiomer of Nutlin-3, Nutlin-3b, on the other hand, did not induce more cell death, with or without reovirus infection (Supplementary Figure S2).

The extent of cell death induced by reovirus was also significantly increased by Nutlin-3a in p53 wild-type U2OS human osteosarcoma and A549 human lung non-small-cell carcinoma cells (Figure 2B and C). Moreover, the percentage of cell death induced by the combination of reovirus and Nutlin-3a was significantly higher than the added value of either treatment alone, suggesting a synergistic cytotoxic effect between Nutlin-3a and reovirus.

To determine whether cell death induced by reovirus and Nutlin-3a co-treatment was a consequence of increased apoptosis, the extent of early apoptosis and cell death was quantified in the presence or absence of the caspase inhibitor I ZVAD. Z-VAD(OMe)-FMK treatment caused significant reduction of apoptotic and dead cells following reovirus infection alone (Figure 2D). Cell death caused by the combination of reovirus and Nutlin-3a was also inhibited by ZVAD, suggesting that p53 accumulation causes increased caspase-dependent apoptosis of reovirus-infected cells (Figure 2D).

### Enhanced apoptosis caused by the combination of reovirus and Nutlin-3a is p53 dependent

**Figure 3:**
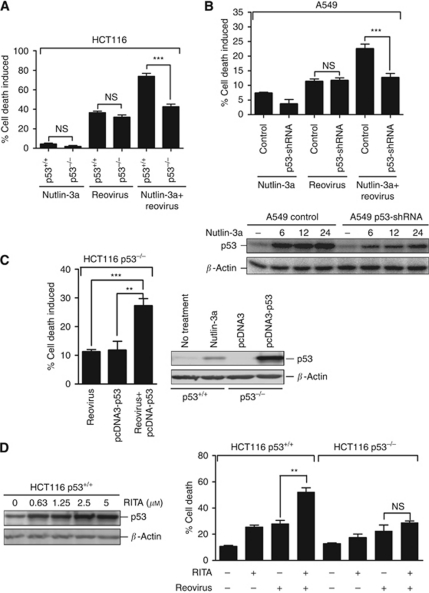
Enhanced cell death induced by the combination of Nutlin-3a and reovirus is dependent on p53. (**A**) The combination of reovirus and Nutlin-3a caused significantly higher levels of cell death in HCT116 p53^+/+^, but not in p53^−/−^ cells. HCT116 p53^+/+^ and p53^−/−^ were either mock infected or infected with reovirus at an MOI of 1. Cells were collected at 24 hpi, subjected to Annexin V and 7-AAD staining and quantified by FACS analysis. (*n*=4, ±s.e.m.). (**B**) Extent of cell death of A549 p53 knockdown cells (A549 p53-shRNA) and non-silencing control cells (A549 control) induced by Nutlin-3a, reovirus (MOI of 100) or the combination of the two reagents (upper panel). p53 levels after Nutlin-3a treatment (lower panel) were determined by western blotting. (**C**) Extent of cell death in reovirus-infected HCT116 p53^−/−^ cells transfected with either p53 expression (pcDNA3-p53) or control pcDNA3 plasmid. HCT116 p53^−/−^ were transfected by pcDNA3-p53 or pcDNA3 plasmids and mock infected or infected with reovirus at an MOI of 1. (**D**) Induction of p53 protein accumulation at different concentrations of RITA (left panel) and percentage of apoptosis in reovirus-infected HCT116 p53^+/+^ and p53^−/−^ cells with or without 0.63 *μ*M RITA treatment (right panel). Student's *t*-test was used to compare two groups of data; NS: not significant; ^**^P<0.001; ^***^P<0.0001.

The enhancement of reovirus-induced cell death by Nutlin-3a was significantly higher in p53^+/+^ than p53^−/−^ HCT116 cells, and was therefore likely mediated directly through p53 rather than potential nonspecific targets in HCT116 cells (Figure 3A). Furthermore, Nutlin-3a had no effects on reovirus-induced apoptosis when p53 was knocked down in A549 cells using p53-specific shRNA (Figure 3B). Conversely, transient overexpression of p53 in HCT116 p53^−/−^ cells using a p53-expression plasmid resulted in significantly higher levels of reovirus-induced apoptosis (Figure 3C). These studies show that p53 accumulation, whether by Nutlin-3a treatment or by transient overexpression, is responsible for enhanced apoptosis of cancer cells induced by reovirus.

Another small molecule, RITA, was also used to induce p53 accumulation. Reactivation of p53 and induction of tumour cell apoptosis inhibits specific interactions between MDM2 and p53 similar to Nutlin-3a, but unlike Nutlin-3a, RITA binds p53 rather than MDM2 (Issaeva *et al*, 2004). As shown in Figure 3D, treatment of HCT116 p53^+/+^ cells with RITA induced accumulation of p53, which significantly enhanced apoptosis compared to reovirus infection alone. The enhancement of apoptosis induced by RITA treatment was not observed in HCT116 p53^−/−^ cells. These results suggest that reovirus oncolysis could be enhanced by RITA, Nutlin-3a or other prospective drugs that promote p53 accumulation.

### Enhanced apoptosis induced by the combination of Nutlin-3a and reovirus requires *Bax* and *p21*

**Figure 4:**
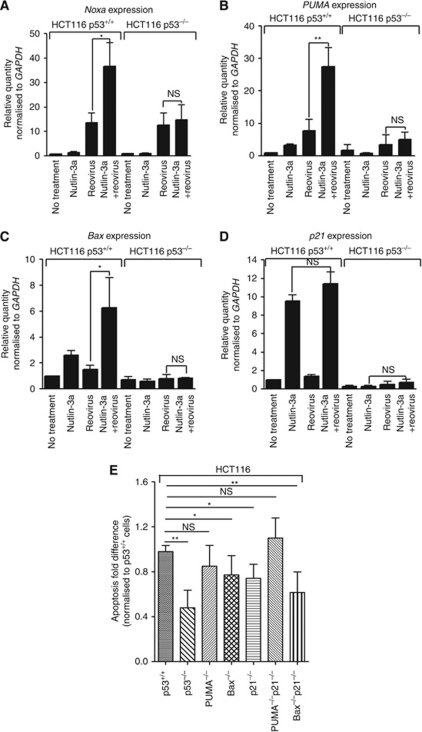
Differential expression levels of p53 target genes (**A**) *Noxa*, (**B**) *PUMA*, (**C**) *Bax* or (**D**) *p21*, induced by reovirus, Nutlin-3a or the combination of the two. Total RNA extracted from HCT116 p53^+/+^ or p53^−/−^, with or without Nutlin-3a treatment or reovirus infection for 24 h (as indicated) was assessed for expression levels of *GAPDH*, *Noxa*, *PUMA*, *Bax* and *p21* by real-time qPCR. Levels of genes were normalised to housekeeping gene *GAPDH*. (**E**) Fold difference of HCT116 knockout cells’ apoptosis induced by the combination of Nutlin-3a and reovirus compared with HCT116 p53^+/+^cells. Percentage of apoptosis induced by Nutlin-3a and reovirus in the knockout cells were normalised to p53^+/+^ cells (*n*=4, ±s.e.m.). Student's *t*-test was used to compare two groups of data; NS: not significant; ^*^P<0.05; ^**^P<0.001.

As the enhancement of apoptosis induced by the combination of Nutlin-3a and reovirus is p53-dependent, we wanted to determine whether expression of p53 target genes was enhanced by the combination of Nutlin-3a and reovirus. RNA samples were collected at 24 hpi and subjected to real-time quantitative polymerase chain reaction (real-time qPCR) using primers specific for *Noxa*, *PUMA*, *Bax* and *p21* (Supplementary Table S1). As expected, Nutlin-3a treatment alone induced increased expression of these p53 target genes *PUMA*, *Bax* and *p21*, although the upregulation of *Noxa* was not as drastic. *PUMA* and *Noxa* were upregulated by reovirus infection alone. When Nutlin-3a and reovirus were combined, expression levels of proapoptotic genes *Noxa*, *PUMA* and *Bax* were further increased in p53^+/+^ cells (Figure 4A–C). As reovirus alone had minimal effect on p21 expression, it is not surprising that the already elevated level of antiapoptotic p21 by Nutlin-3a treatment alone was not further enhanced by the combination treatment (Figure 4D). Therefore, combined Nutlin-3a and reovirus treatment had a more pronounced effect on the expression of proapoptotic genes than proarrest genes at 24 hpi when apoptosis was the desirable outcome.

To further characterise p53 target genes that were important for the enhancement of apoptosis induced by the combination of Nutlin-3a and reovirus, we obtained a panel of knockout cells derived from HCT116 (p53^+/+^) from Dr Vogelstein (Supplementary Figure S3A). These cells were treated with Nutlin-3a, reovirus or the combination of the two and apoptosis levels were assayed as described before. Status of *PUMA* or *Noxa* did not seem to affect the enhancement of apoptosis induced by Nutlin-3a and reovirus (Figure 4E and Supplementary Figure S3C, right panel). Interestingly, although levels of cell death induced by reovirus alone did not significantly vary among all the knockout cells compared to p53^+/+^ cells (Supplementary Figure S3B), levels of apoptosis induced by the combination of Nutlin-3a and reovirus were significantly decreased in Bax^−/−^ and p21^−/−^ cells. A further decrease in apoptosis level was observed in Bax^−/−^p21^−/−^ cells (Figure 4E), indicating that both Bax and p21 were required for the p53-dependent enhancement of apoptosis induced by Nutlin-3a and reovirus. The upregulation of proarrest p21 by Nutlin-3a might have blocked apoptosis at earlier times to allow reovirus to replicate efficiently, whereas enhanced Bax expression by Nutlin-3a and reovirus combined at later times would promote apoptosis for virus release.

### Apoptosis induced by Nutlin-3a and reovirus requires NF-*κ*B activation

**Figure 5:**
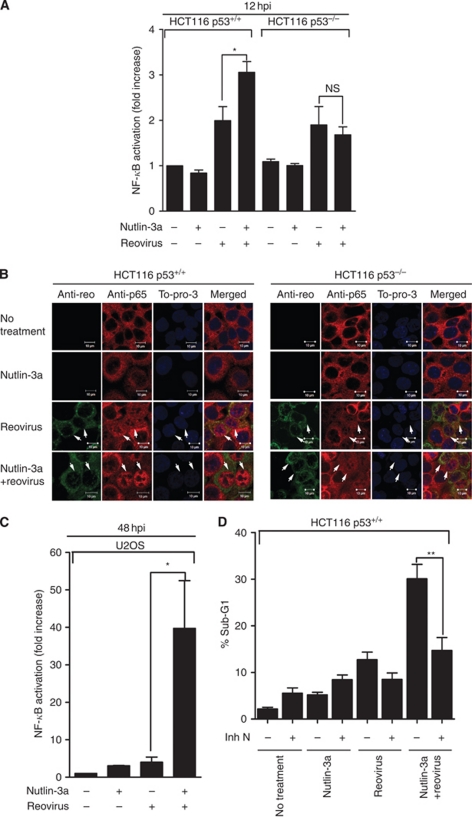
Cell death induced by Nutlin-3a and reovirus requires NF-*κ*B activation. Levels of NF-*κ*B transcription activity after reovirus infection in HCT116 p53^+/+^ cells (**A**) and U2OS cells (**C**) (*n*=4, ±s.e.m.). HCT116 cells were infected at an MOI of 1 and lysates were collected at 12 hpi. U2OS cells were infected at an MOI of 500 and lysates were collected at 48 hpi. (**B**) Nutlin-3a treatment enhanced levels of NF-*κ*B p65 nuclear translocation. Arrows point to infected cells. (**D**) NF-*κ*B activation inhibitor N (InSolution NF-*κ*B activation inhibitor at 1 : 100 000 dilution) treatment reduced the level of cell death induced by the combination of reovirus and Nutlin-3a in p53^+/+^ HCT116 cells. Cell death was determined by quantifying the sub-G1 population of PI-stained cells. Student's *t*-test was used to compare two groups of data; NS: not significant; ^*^P<0.05; ^**^P<0.001.

**Figure 6:**
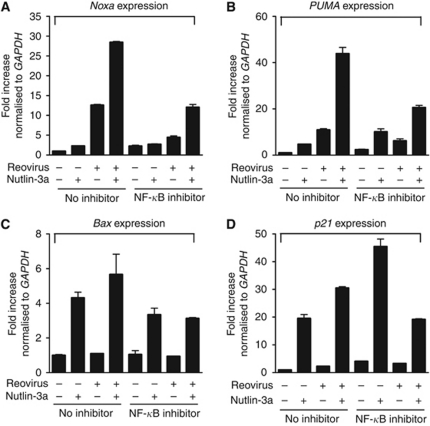
Upregulation of p53 target genes (**A**) *Noxa*, (**B**) *PUMA*, (**C**) *Bax* and (**D**) *p21* induced by the combination of Nutlin-3a and reovirus was inhibited by NF-*κ*B inhibitor N. HCT116 p53^+/+^ cells were pretreated with NF-*κ*B inhibitor N for 1 h before Nutlin-3a treatment. At 6 hpi, cells were infected by reovirus at an MOI of 1. RNA samples were collected at 24 hpi and subjected to RNA purification, cDNA synthesis and real-time PCR (representative of two respective experiments, ±s.e.m.).

It has previously been demonstrated that reovirus infection causes NF-*κ*B activation. Furthermore, NF-*κ*B was shown to be necessary for reovirus-induced apoptosis, as inhibition of NF-*κ*B activation through disruption of I*κ*B degradation caused diminished reovirus-induced apoptosis. Cells deficient for either p50 or p65 subunits of NF-*κ*B were also found to be resistant to reovirus infection (Connolly *et al*, 2000; Clarke *et al*, 2003). We found that reovirus infection alone caused activation of NF-*κ*B transcription in both p53^+/+^ and p53^−/−^ cells using a dual-luciferase assay, whereas Nutlin-3a alone had negligible effect on the activation of NF-*κ*B (Figure 5A and C and Supplementary Figure S4A and B). When cells were challenged by the combination of reovirus and Nutlin-3a, however, the level of NF-*κ*B transcription activation was further increased in p53^+/+^ cells (wild-type HCT116 and U2OS cells), but not in p53^−/−^ HCT116 cells, indicating that the enhancement of NF-*κ*B activation caused by Nutlin-3a and reovirus was p53-dependent. In accordance with our results shown above, immunostaining of NF-*κ*B subunit p65 (Figure 5B) showed that Nutlin-3a itself did not affect p65 localisation; reovirus infection clearly induced nuclear translocation of p65 in both p53^+/+^ and p53^−/−^ cells. However, the addition of Nutlin-3a significantly enhanced the nuclear localisation of p65 in p53^+/+^ cells, but not in p53^−/−^ cells, further confirming that the combination of reovirus and Nutlin-3a could induce significantly higher level of NF-*κ*B activation and the enhancement is p53-dependent. Furthermore, when NF-*κ*B activation was prevented by NF-*κ*B activation inhibitors, evidenced by the blockage of p65 nuclear translocation (Supplementary Figure S4C and D), cell death of p53^+/+^ HCT116 cells cotreated with reovirus and Nutlin-3a was significantly reduced (Figure 5D and Supplementary Figure S4D). These results suggest that the cytotoxicity caused by the combination of reovirus and Nutlin-3a is mediated through activation of NF-*κ*B signalling pathways. We further determined the RNA levels of representative p53 target genes with or without the treatment of NF-*κ*B inhibitor **N**. As shown in Figure 6, inhibition of NF-*κ*B clearly suppressed the upregulation of these genes with the treatment of reovirus and Nutlin-3a. Therefore, the p53-dependent NF-*κ*B activation likely plays a role in controlling the upregulation of p53 target genes, indicating a link between NF-*κ*B activation and the enhancement of apoptosis in Nutlin-3a- and reovirus-treated cells.

Clarke *et al* (2000) showed that reovirus-induced apoptosis requires proapoptotic signalling through extracellular TRAIL and its receptors DR4 and DR5 in certain cell types. As Nutlin-3 can upregulate the expression and externalisation of TRAIL receptor DR5 (Hori *et al*, 2009), the involvement of TRAIL was investigated. Anti-TRAIL antibody and competitive soluble DR4 and DR5 ligands were used to subdue the extrinsic TRAIL response, and effectively prevented cell death caused by exogenously added TRAIL (Supplementary Figure S5A). However, anti-TRAIL antibody and competitive DR4- and DR5-soluble ligands did not prevent apoptosis induced by either reovirus or the combination of reovirus and Nutlin-3a (Supplementary Figure S5B). Therefore, the extrinsic TRAIL-mediated pathway does not seem to play a major role in the enhancement of apoptosis induced by reovirus and Nutlin-3a.

### Combination of Nutlin-3a significantly increases reovirus-induced cytotoxicity to cancer cells and promotes virus dissemination

**Figure 7:**
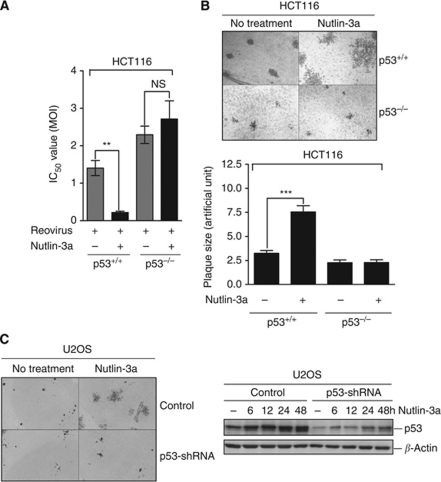
Nutlin-3a significantly enhanced both reovirus-induced cytotoxicity and reovirus dissemination in HCT116 p53^+/+^ cells. (**A**) IC_50_ values of reovirus in HCT116 p53^+/+^ or p53^−/−^ cells in the presence or absence of 5 *μ*M Nutlin-3a at 48 hpi. (**B** and **C**) Immunohistochemical staining of reovirus-infected HCT116 cells (**B**) and U2OS cells (**C**) in the presence or absence of Nutlin-3a treatment. (**B**, lower panel) Quantification of the plaque size on reovirus-infected HCT116 cells (plaque number *n*=14, ±s.e.m.). Cells were fixed at 7-day post-infection and proceed to staining using anti-reovirus antibody, shown as the representative of three respective experiments. Student's *t*-test was used to compare two groups of data; NS: not significant; ^**^P<0.001; ^***^P<0.0001.

As the combination of Nutlin-3a and reovirus is significantly more cytotoxic to cancer cells, we wanted to explore the possibility of using reovirus and Nutlin-3a as a combination therapy for cancers with wild-type p53. To investigate whether Nutlin-3a could improve the efficacy of reovirus, different doses of reovirus were used to infect HCT116 cells in the presence or absence of 5 *μ*M Nutlin-3a and IC_50_ of reovirus was calculated accordingly. As shown in Figure 7A, Nutlin-3a drastically reduced the IC_50_ of the input reovirus (from 1.4 to 0.22 MOI) in HCT116 p53^+/+^ cells while having negligible effect on p53^−/−^ cells. Therefore, the combination of reovirus and Nutlin-3a has the potential as a combination therapy in p53^+/+^ cancer.

In addition to cytotoxicity, viral oncolysis relies on efficient cell-to-cell spread of virus to overcome the growing tumour. The increased cytotoxicity of reovirus-infected cells and augmented release of new progeny virions by Nutlin-3a suggests that Nutlin-3a could facilitate both cytotoxicity and rapid viral dissemination among tumour cells. Virus cell-to-cell spread can be easily visualised with a virus plaque assay, where agar overlay restricts virus infection only among neighbouring cells. The size of the plaque reflects the extent of virus dissemination over several rounds of replication, release and re-infection (Marcato *et al*, 2007). As shown in Figure 7B, reovirus plaques formed on Nutlin-3a-treated HCT116 p53^+/+^ cells were significantly bigger than the ones on vehicle-treated cells, indicating that Nutlin-3a could enhance the spread of reovirus to the neighbouring cells. The enlargement of reovirus plaques by Nutlin-3a was also observed in U2OS control cells, but not the p53-shRNA knockdown cells (Figure 7C). Taken together, these results suggest that Nutlin-3a co-treatment confers two advantages important for viral oncolysis; Nutlin-3a supports increased killing of p53^+/+^ cancer cells and subsequently also permits rapid reovirus dissemination.

## Discussion

Our study is the first to report that the combination of drugs that cause p53 accumulation and reovirus can induce enhanced cytotoxicity in cancer cells. Although the status of p53 in HCT116 tumour cells does not affect reovirus production, the intact function of p53 in cancer cells can be utilised to promote reovirus-mediated, caspase-dependent apoptosis of cancer cells.

Recently, Kim *et al* (2010) reported that lack of p53 in certain cells render them susceptible to reovirus infection. Our data showed that there was no significant difference between the HCT116 p53^+/+^ and p53^−/−^ cells in reovirus production within at least the first round of reovirus infection (18 hpi; Figure 1A) and the level of apoptosis induced by reovirus was not significantly different between p53^+/+^ and p53^−/−^ HCT116 cells or between A549 control and p53 knockdown cells (Figure 3A and B). Therefore, p53-dependent resistance of tumour cells might be cell type-dependent. Moreover, for less susceptible cancer cells (e.g., U2OS), when combined with reovirus, Nutlin-3a treatment clearly induced higher levels of apoptosis and reovirus spread (Figure 2B and 7C). Therefore, our results indicate that irrespective of the effects of wild-type p53 on reovirus infection, p53 *accumulation* with Nutlin-3a treatment ultimately promotes reovirus-mediated cancer cell killing.

The NF-*κ*B transcription factor plays an important role in regulating apoptosis and appears to be involved in a mechanism by which reovirus and Nutlin-3a synergise for increased cancer cell killing. NF-*κ*B was clearly induced by the combination of reovirus and Nutlin-3a in a p53-dependent manner, and induction of NF-*κ*B was required for the full cytotoxic potential of reovirus and Nutlin-3a co-treatment. It was previously reported that NF-*κ*B is essential for p53-dependent apoptosis and that p53 can enhance the activation of NF-*κ*B (Ryan *et al*, 2000, 2004). In our study, we showed that certain p53 target genes were upregulated by the combination of Nutlin-3a and reovirus, among which, *p21* and *Bax* seem to play a bigger role in promoting cell death induced by the combination of the two agents, as p21^−/−^, Bax^−/−^ and Bax^−/−^p21^−/−^ cells had a significantly decreased level of apoptosis. Moreover, inhibition of NF-*κ*B activation by treatment of NF-*κ*B inhibitor was shown to suppress the upregulation of p53 target genes with the treatment of reovirus and Nutlin-3a, further confirming the link between the enhancement of NF-*κ*B activation and the increase in cytotoxicity induced by the combination of p53 accumulation and reovirus infection.

The majority of existing chemotherapy reagents function by causing damage to cells with the premise that highly dividing cancer cells are more prone to the cytotoxic effects. Unfortunately, chemotherapy reagents also damage normal cells and result in various side effects that reduce the quality of life of cancer patients. Attempts are ongoing to reduce unwanted side effects of cancer therapies through alternative cancer treatments and/or through combinations of distinct cancer therapeutics at reduced dosages. Concurrently, reovirus and other oncolytic viruses are being tested as cancer therapeutics. Reovirus is a promising anticancer therapy and phase I clinical trials of reolysin showed limited toxicity towards patients (Forsyth *et al*, 2008; Vidal *et al*, 2008; Harrington *et al*, 2010). Nutlin-3a was also found to exhibit no pathological effect in *in vivo* murine studies (Vassilev *et al*, 2004). According to our results, the combination of the two low-toxic reagents (reovirus and Nutlin-3a) could be an alternative way of inducing cancer regression without exposing cancer patients to unwanted side effects.

Previous studies that combining reovirus with chemotherapeutics did not address that role of p53 in the outcome of the combination (Pandha *et al*, 2009; Sei *et al*, 2009). Our results, however, raise the possibility that status of p53 in cancer cells might have effects on combination of chemotherapy drugs with reovirus. Determining whether combination of chemotherapy drugs with reovirus have differential effect on p53^+/+^ and p53^−/−^ would be important to direct personalised cancer therapy in the future.
