# Supplementary material for: Stabilisation of p53 enhances reovirus-induced apoptosis and virus spread through p53-dependent NF-κB activation
Source: Br J Cancer. 2011 Aug 23;105(7):1012–22. doi: 10.1038/bjc.2011.325 (PMC3185941; doi:10.1038/bjc.2011.325)
Supplement: Supplementary Figures S1–S5 [file bjc2011325x1.ppt]

## Slide 1
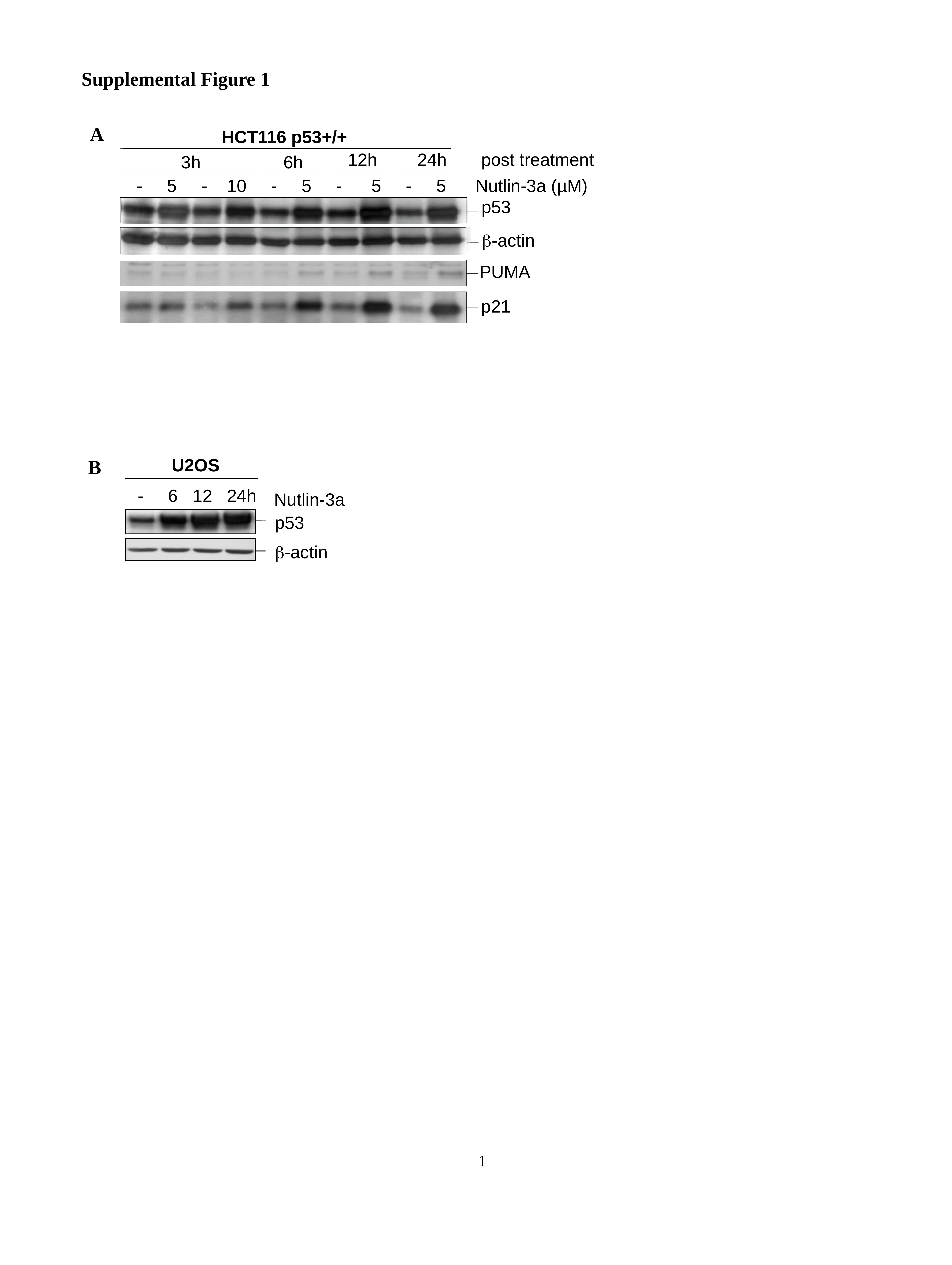

Supplemental Figure 1
A
HCT116 p53+/+
12h
 24h post treatment
3h
6h
 - 5 - 10 - 5 - 5 - 5 Nutlin-3a (µM)
p53
β-actin
PUMA
p21
U2OS
- 6 12 24h
Nutlin-3a
p53
β-actin
B
1

## Slide 2
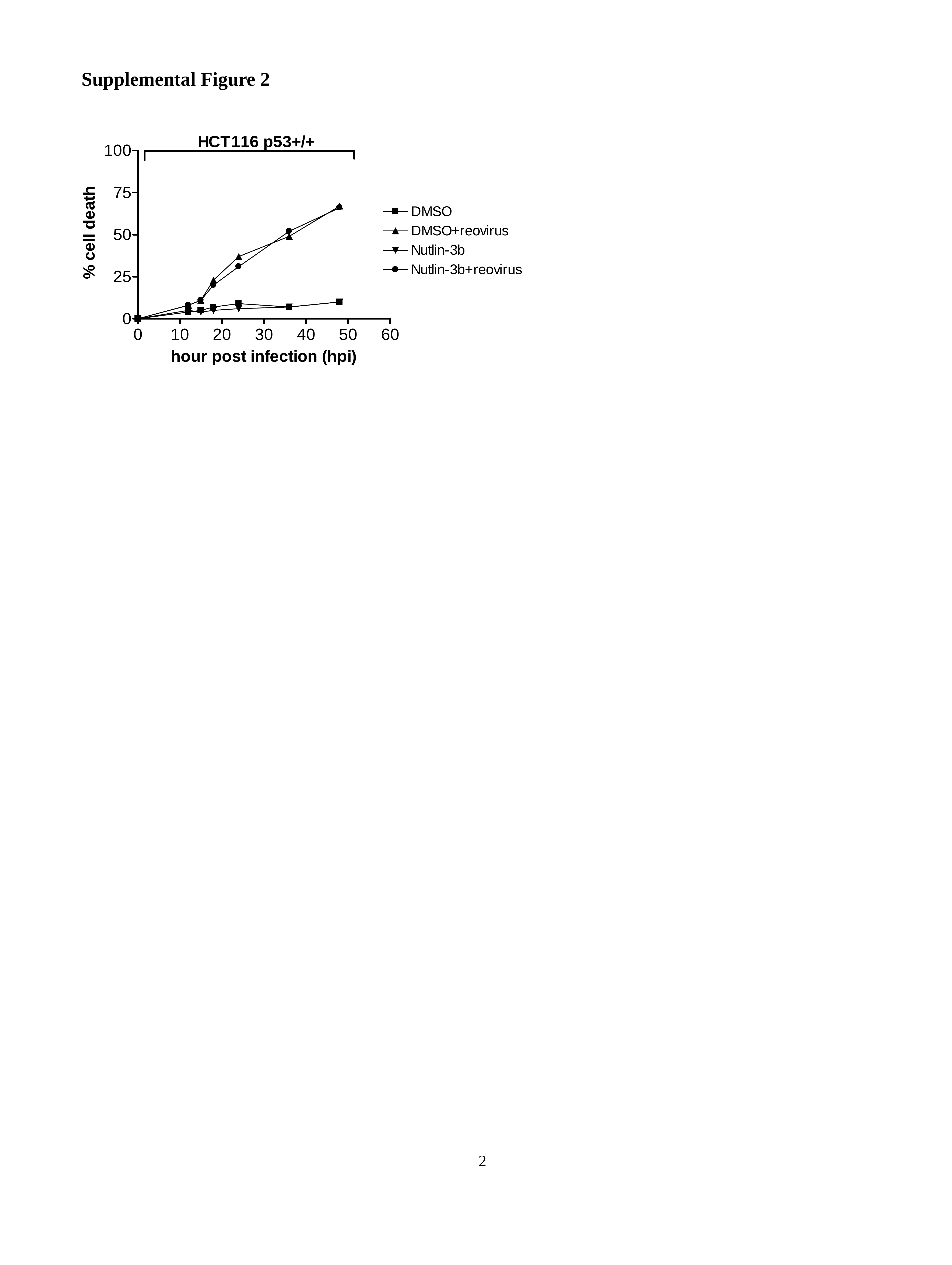

Supplemental Figure 2
2

## Slide 3
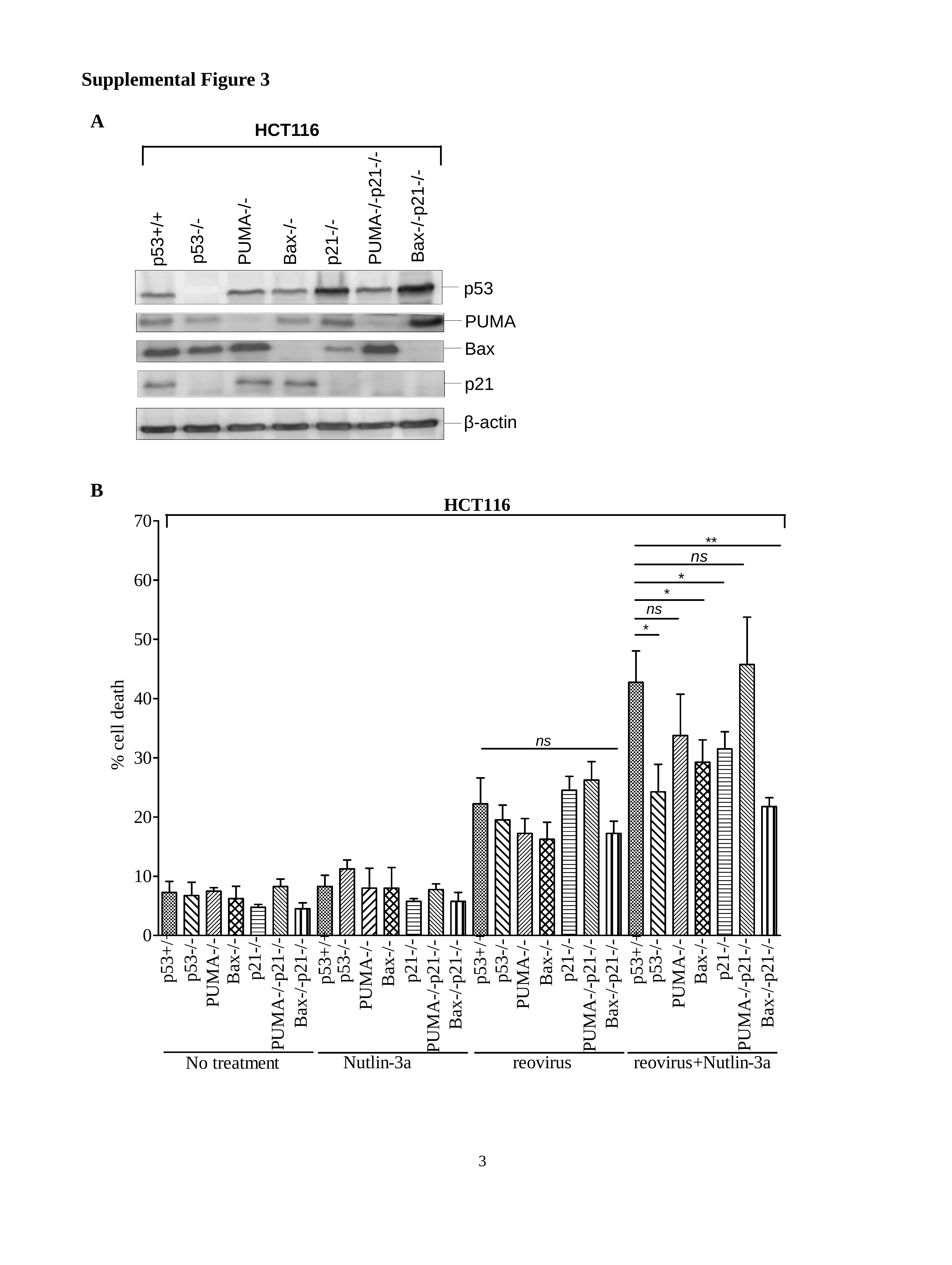

Supplemental Figure 3
A
HCT116
PUMA-/-p21-/-
Bax-/-p21-/-
PUMA-/-
p53+/+
p53-/-
Bax-/-
p21-/-
p53
PUMA
Bax
p21
β-actin
B
3

## Slide 4
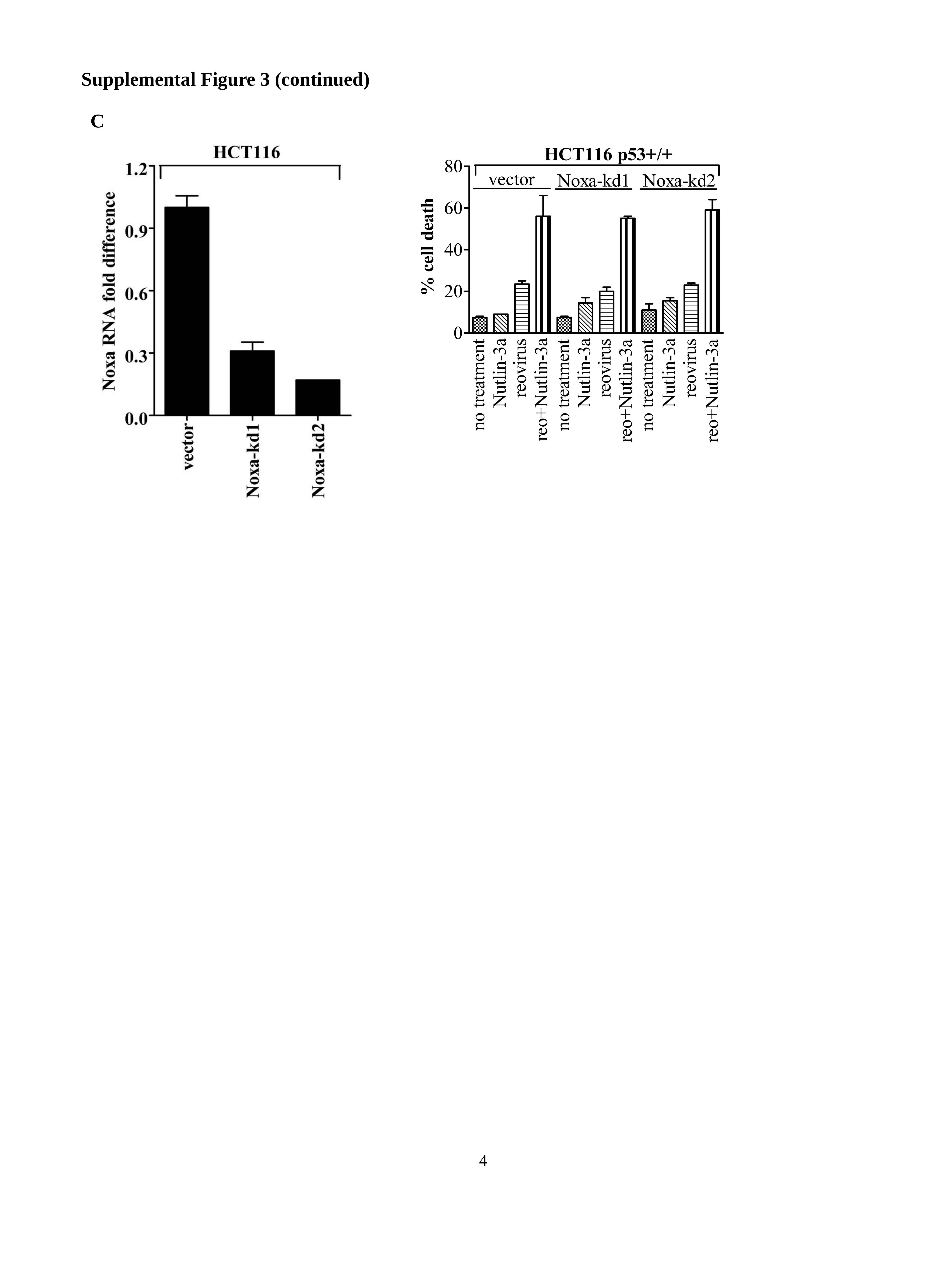

Supplemental Figure 3 (continued)
C
4

## Slide 5
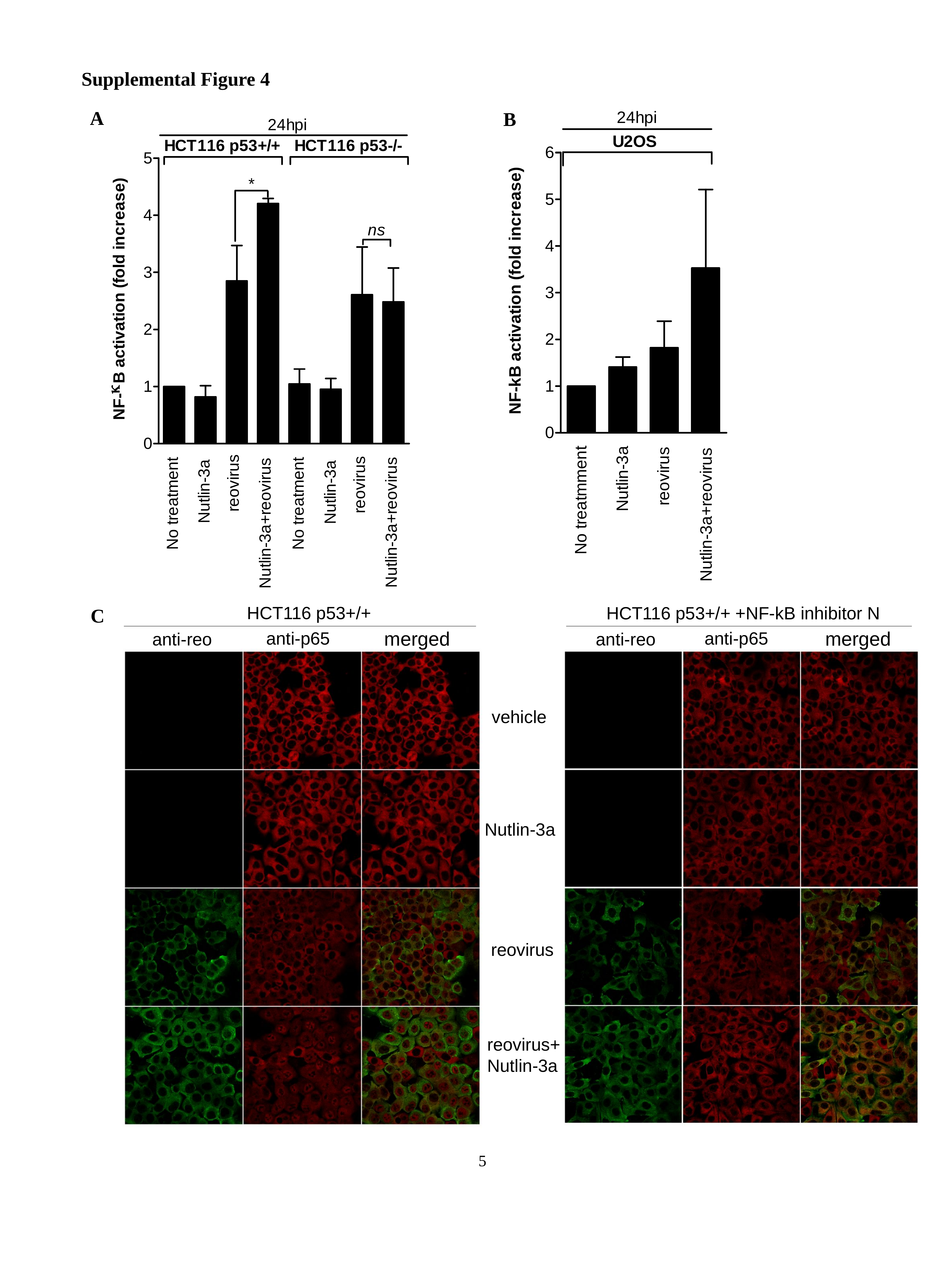

Supplemental Figure 4
A
B
HCT116 p53+/+
HCT116 p53+/+ +NF-kB inhibitor N
C
merged
merged
anti-p65
anti-p65
anti-reo
anti-reo
vehicle
Nutlin-3a
reovirus
reovirus+
Nutlin-3a
5

## Slide 6
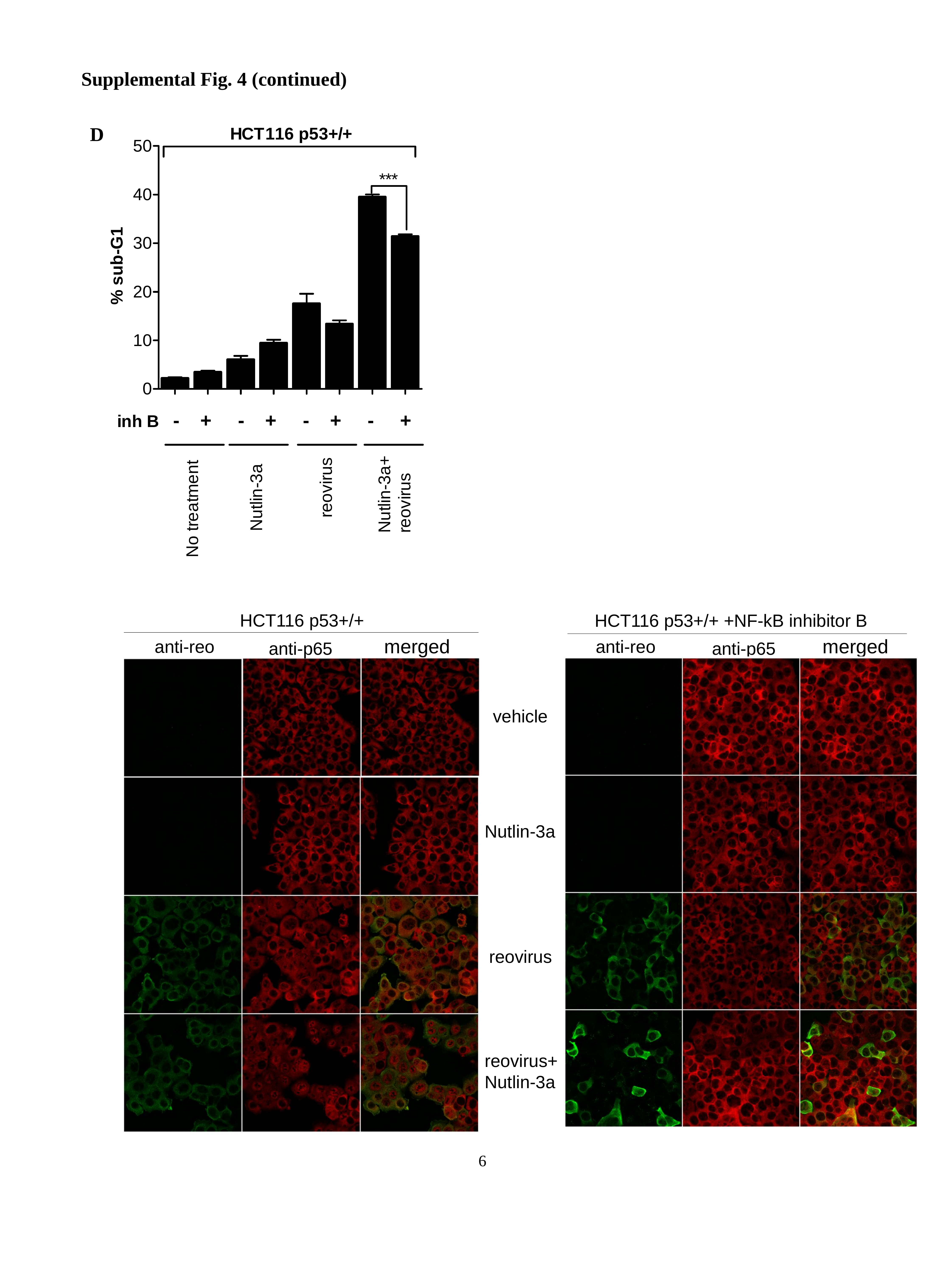

Supplemental Fig. 4 (continued)
D
HCT116 p53+/+
HCT116 p53+/+ +NF-kB inhibitor B
merged
merged
anti-reo
anti-reo
anti-p65
anti-p65
vehicle
Nutlin-3a
reovirus
reovirus+
Nutlin-3a
6

## Slide 7
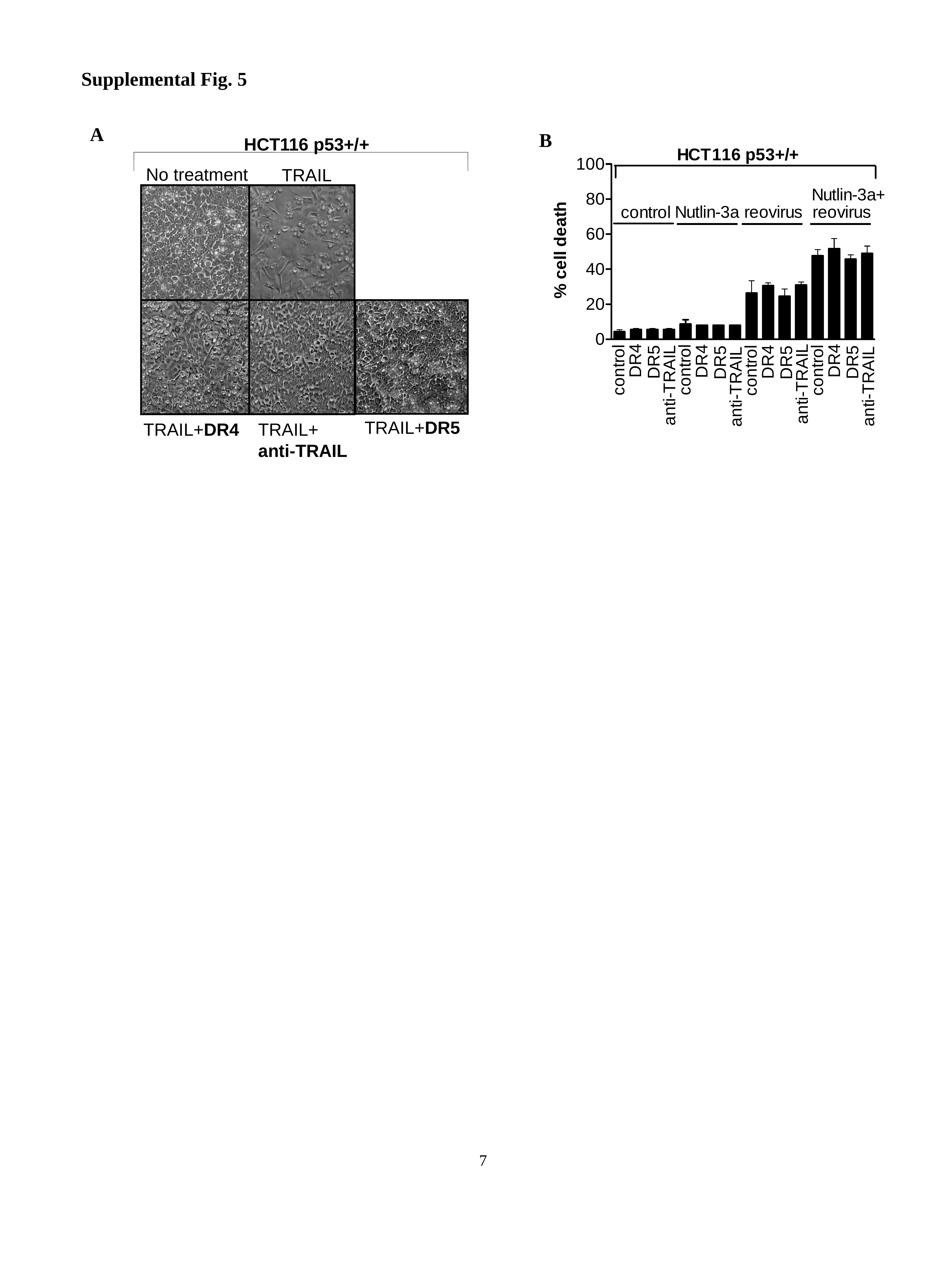

Supplemental Fig. 5
A
B
HCT116 p53+/+
No treatment
TRAIL
TRAIL+DR5
TRAIL+DR4
TRAIL+
anti-TRAIL
7
