# Supplementary material for: Stabilisation of p53 enhances reovirus-induced apoptosis and virus spread through p53-dependent NF-κB activation
Source: Br J Cancer. 2011 Aug 23;105(7):1012–22. doi: 10.1038/bjc.2011.325 (PMC3185941; doi:10.1038/bjc.2011.325)
Supplement: Supplementary Figure Legends [file bjc2011325x2.doc]

## Supplemental Fig. 1. (A) p53 accumulation after Nutlin-3a treatment in p53+/+ cells. p53+/+ HCT116 cells were treated with Nutlin-3a and were collected at different time-points and subjected to western blotting using p53, p21 and PUMA-specific antibodies. (B) p53 levels in U2OS cells after Nutlin-3a treatment (6, 12 and 24 hour post treatment) were determined by western blotting.

**Supplemental Fig. 2.** Effect of Nutlin-3b (inactive enantiomer of Nutlin-3) on mock or reovirus-infected HCT116 p53+/+ cells. Cells were infected at MOI of 1 with or without pre-treatment using Nutlin-3b or DMSO control. Cells were harvested at 12, 15, 18, 24, 36 and 48hpi and stained with Annexin V and 7-AAD to determine levels of apoptosis induced by reovirus or the combination of reovirus and Nultin-3b.

**Supplemental Fig. 3.** (A) Western blot analysis for HCT116 cells (p53+/+, p53-/-, PUMA-/-, Bax-/-, PUMA-/-p21-/- and Bax-/-p21-/-). (B) Levels of cell death induced by Nutlin-3a, reovirus or the combination of the two in HCT116 knockout cells. (C) Noxa status did not significantly affect apoptosis level of HCT116 cells. RNA levels of Noxa were determined by real-time PCR (left panel) and vector control cells and Noxa knockdown (kd) cells were subjected to treatment of Nutlin-3a, reovirus or the combination of the two. Levels of cell death were determined by Annexin V and 7-AAD staining (right panel, n=2, *±SEM*). Lentivirus vector pLKO.1-puro (control) and Noxa-kd plasmids were purchased from Sigma Aldrich (TRCN0000150554 for Noxa-kd1 and TRCN0000150555 for Noxa-kd2).

## Supplemental Fig. 4. Levels of NF-κB transcription activity after reovirus infection in HCT116 p53+/+ cells (A) and U2OS cells (B) (*n=4, ±SEM*). HCT116 cells were infected at MOI of 1 and lysates were collected at 24hpi (left panel); U2OS cells were infected at MOI of 500 and lysates were collected at 24hpi (right panel). (C) NF-κB p65 translocation induced by reovirus or a combination of reovirus and Nutlin-3a was blocked by treatment of NF-κB activation inhibitor N (Cat# 481407). Cells were fixed with 4% paraformaldehyde at 12hpi. (D) NF-κB activation inhibitor B (Cat# 196871) (InSolution^TM^ BAY 11-7082 at 1:10,000 dilution) treatment reduced the level of NF-κB p65 translocation (upper panel) as well as cell death (lower panel) induced by the combination of reovirus and Nutlin-3a in p53+/+ HCT116 cells at 24hpi. Cells were fixed with 4% paraformaldehyde at 12hpi for immunostaining. Cell death was determined by quantifying the sub-G1 population of PI-stained cells.

## Supplemental Fig. 5. (A) Effect of soluble DR4 (100ng/mL), DR5 (100ng/mL) and anti-TRAIL (30µg/mL) antibody towards TRAIL-induced cell cytotoxicity (TRAIL: 20ng/mL). (B) Soluble DR4, DR5 and anti-TRAIL antibody did not reduce levels of apoptosis induced by the combination of Nutlin-3a and reovirus. Competitive soluble DR4 or DR5 ligands (500 μg/mL) or anti-TRAIL antibody (300 μg/mL) were used to treat HCT116 p53+/+ cells one hour before Nutlin-3a treatment. Apoptosis was determined at 24hpi with Annexin V and 7-AAD staining.
