# Supplementary material for: Stabilisation of p53 enhances reovirus-induced apoptosis and virus spread through p53-dependent NF-κB activation
Source: Br J Cancer. 2011 Aug 23;105(7):1012–22. doi: 10.1038/bjc.2011.325 (PMC3185941; doi:10.1038/bjc.2011.325)
Supplement: Supplementary Table 1 [file bjc2011325x3.doc]

## Supplemental Table 1. Specific primers for detecting human Noxa, PUMA, Bax, p21 and GAPDH in real-time quantitative PCR analysis.

| GAPDH-rt-fwd | GAGTCAACGGATTTGGTCGT |
| --- | --- |
| GAPDH-rt-rev | TTGATTTTGGAGGGATCTCG |
| p21-rt-fwd | GACACCACTGGAGGGTGACT |
| p21-rt-rev | CAGGTCCACATGGTCTTCCT |
| PUMA-rt-fwd | GACGACCTCAACGCACAGTA |
| PUMA-rt-rev | CACCTAATTGGGCTCCATCT |
| Bax-rt-fwd | TCAGGATGCGTCCACCAAGAAG |
| Bax-rt-rev | TGTGTCCACGGCGGCAATCATC |
| Noxa-rt-fwd | CTTGGAAACGGAAGATGGAA |
| Noxa-rt-rev | CACAGTAGGCCAGCGGTAAT |
